# Methodological Shortcomings in Meta‐Analysis of Wood Dust Exposure and Laryngeal Cancer

**DOI:** 10.1002/cam4.71064

**Published:** 2025-07-17

**Authors:** Lidwien A. M. Smit

**Affiliations:** ^1^ Institute for Risk Assessment Sciences Utrecht University Utrecht the Netherlands

I read with great interest the recent meta‐analysis by Meng et al. [1] on occupational wood dust exposure and laryngeal cancer, published in Cancer Medicine. This topic is highly significant, particularly for informing the development and implementation of effective occupational health policies. However, I have substantial concerns regarding several methodological aspects that may compromise the reliability of its findings.

Notably, an earlier meta‐analysis by Paget‐Bailly et al. [2], published in 2012, already includes 18 of the 19 studies cited in the current article, rendering this new analysis largely redundant, with only one additional study by Langevin et al. [3] published in 2013. Furthermore, the case and control numbers for the Langevin et al. study are reported incorrectly by Meng et al.; the correct figures should be 159 cases and 1193 controls, not 213 and 1442 [3]. Additionally, the odds ratio (OR) used in the meta‐analysis is based on ‘each decade of occupational exposure to sawdust’, whereas the OR for ‘ever exposure’ would be more comparable to other OR included in the meta‐analysis.

Using a random‐effects model and risk estimates from the 19 studies, Meng et al. report an overall OR for the association between wood dust exposure and laryngeal cancer of 1.11 (95% CI: 0.93–1.33). Surprisingly, in a subgroup analysis, the authors report an OR of 1.14 (95% CI: 1.01–1.25) for studies with more than 200 cases and 1.29 (95% CI: 1.00–1.66) for studies with fewer than 200 cases. However, upon recalculation using a random‐effects model and the data presented in Table 1 of Meng et al. [1], the ORs should be 1.03 (95% CI: 0.85–1.25) for studies with more than 200 cases and 1.37 (95% CI: 0.94–2.04) for those with fewer than 200 cases. It is possible the authors used fixed‐effects models for these subgroups, but given the moderate to high heterogeneity (*I* ^2^ > 40%), random‐effects models would be more appropriate.

Moreover, the study lacks sufficient detail on how wood dust exposure was defined and assessed across studies, which is critical for interpretation and for ensuring consistency in the results. While the Newcastle‐Ottawa Scale is mentioned as a quality control measure, it is neither shown nor applied in the paper, further undermining the study's methodological rigor.

These issues are concerning, as robust and accurate meta‐analyses are essential for guiding public and occupational health decisions, but unfortunately, this study falls short in several key areas. As it stands, this meta‐analysis fails to provide reliable evidence supporting an association between wood dust exposure and laryngeal cancer.

## Author Contributions

**Lidwien A. M. Smit:** conceptualization, writing – original draft, investigation.

## Conflicts of Interest

The author declares no conflicts of interest.

## Data Availability

The author has nothing to report.
